# Adiponectin, Retinoic Acid Receptor Responder 2, and Peroxisome Proliferator-Activated Receptor-*γ* Coativator-1 Genes and the Risk for Obesity

**DOI:** 10.1155/2017/5289120

**Published:** 2017-08-29

**Authors:** Ana Carolina Proença da Fonseca, Alan Cleveland Ochioni, Raisa da Silva Martins, Verônica Marques Zembrzuski, Mario Campos Junior, Vivianne Galante Ramos, João Regis Ivar Carneiro, José Firmino Nogueira Neto, Pedro Hernan Cabello, Giselda Maria Kalil Cabello

**Affiliations:** ^1^Human Genetic Laboratory, Oswaldo Cruz Institute/FIOCRUZ, Rio de Janeiro, RJ, Brazil; ^2^Human Genetic Laboratory, Grande Rio University, Rio de Janeiro, RJ, Brazil; ^3^Clementino Fraga Filho University Hospital, Rio de Janeiro, RJ, Brazil; ^4^Department of Pathology and Laboratory, Rio de Janeiro State University, Rio de Janeiro, RJ, Brazil

## Abstract

Obesity is the most common nutritional disorder. This disease is a multifactorial disease influenced by environmental and genetic factors. This study investigated the relationship between common variants of adiponectin (*ADIPOQ*), retinoic acid receptor responder 2 (*RARRES2*), and peroxisome proliferator-activated receptor-*γ* coativator-1 (*PPARGC1*) and obesity-related traits and susceptibility. A total of 167 individuals with obesity and 165 normal-weight subjects were recruited. Genotype frequencies of rs182052 in *ADIPOQ* differed significantly between the groups. Genotype AA was observed at a higher frequency in case than in control subjects. Association analysis showed that the A allele was a risk factor for obesity. This polymorphism was associated with body weight, body mass index (BMI), and waist circumference. After stratification by BMI, eutrophic individuals with AA or AG genotypes had higher body weights and waist circumferences than those with GG genotypes. In the case group, no associations were observed, except for stratified subjects with morbid obesity that exhibited a progressive increase of body weight, BMI, and waist circumference when rs182052 A was present. No associations were observed between SNPs in *RARRES2* and *PPARGC1* and obesity or any other studied variables. The rs182052 polymorphism in *ADIPOQ* is associated with a higher risk for obesity and obesity-related parameters.

## 1. Introduction

**Figure 1:**
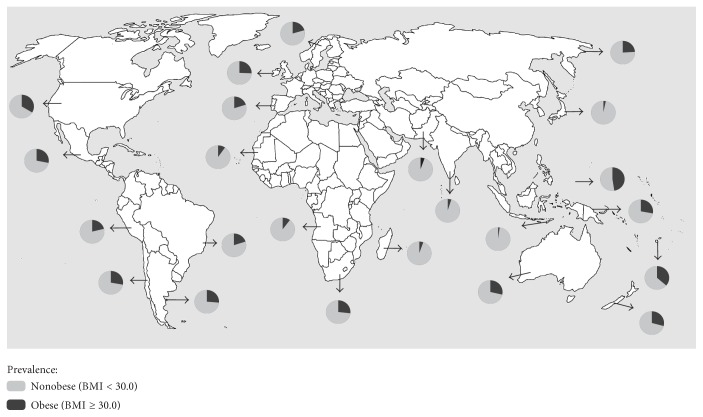
Prevalence of obesity. Source: World Health Organization 2015.

Obesity is a major public health problem worldwide [1]. This disease has an increasing prevalence in most countries, affecting populations from developed and developing countries (Figure 1). Recent data from the World Health Organization estimated that the prevalence of obesity more than doubled between 1980 and 2014 in the world. Currently, more than 1.9 billion adults have overweight and at least 600 million have obesity [2]. In Brazil, excess body weight affects half of all men and women, and data show that 20% of individuals have obesity [2, 3]. Furthermore, the worldwide prevalence of obesity continues to increase at an alarming rate, resulting in nearly 3 million deaths every year [4].

In recent years, the population's weight gain has been influenced heavily by changes in eating and lifestyle that have increased the consumption of hypercaloric foods and reduced energy expenditure [5]. Even today, obesity is commonly portrayed stereotypically as a problem of gluttonous behavior. However, there are clear differences in susceptibility among different individuals or communities. This observation suggests that the control of body weight has a multifactorial nature and that genetic differences between individuals could play an important role in obesity risk within a given environment [6, 7].

Over the last two decades, researchers have made a continuous effort to identify genes and variants that predispose individuals to common forms of obesity. Over 120 candidate genes have been identified. However, less than 20% have been replicated by five or more other studies [8]. Among the confirmed genes are adiponectin (*ADIPOQ*), retinoic acid receptor responder 2 (*RARRES2*), and peroxisome proliferator-activated receptor-*γ* coativator-1 (*PPARGC1*).

*ADIPOQ* encodes adiponectin, a major adipocyte secretory protein. This protein acts in numerous tissues, regulating lipid and glucose metabolism, fat oxidation in the skeletal muscle and liver, and decreasing hepatic glucose production [9, 10]. *RARRES2* encodes the adipokine chemerin that reportedly plays a role in adipogenesis and adipocyte metabolism [11]. *PPARGC1* is a member of a transcriptional coactivator family [12]. This coactivator protein regulates adaptive thermogenesis, mitochondrial fatty acid oxidation, hepatic gluconeogenesis, and adipogenesis, as well as glucose uptake and lipid metabolism [13–15].

These genes are responsible for encoding adipokines involved in the regulation of various metabolic pathways and are important for maintaining body energy homeostasis. Therefore, genetic variations may affect protein functions or gene expression efficiency and contribute to several pathophysiological conditions. The aim of the present study was to test the impact of common genetic variations in *ADIPOQ*, *RARRES2*, and *PPARGC1* on obesity-related traits and susceptibility in a sample of adult subjects from Rio de Janeiro, Brazil.

## 2. Materials and Methods

### 2.1. Subjects

This case-control cross-sectional study comprised 332 adult subjects, aged 18 to 65 years, from the state of Rio de Janeiro in southeastern Brazil. Exclusion criteria were pregnancy, lactation, and the use of medication to lose weight. For this study, 167 individuals with obesity (BMI ≥ 30.0) and 165 subjects with normal weight (18.5 ≤ BMI ≤ 24.9) were selected. All people with obesity were recruited from a nongovernmental organization, the Rescue Group to Self-Esteem and Citizenship of the Obese (GRACO). The controls were volunteers from public hospitals of Rio de Janeiro, Brazil. The subject characteristics (anthropometric and biological) are shown in Table 1. All participants provided written consent to participate in this study, and the protocol was performed according to the Declaration of Helsinki (1964) and approved by Ethics Committee of the Oswaldo Cruz Foundation.

Anthropometrical characteristics, including height, weight, and waist and hip circumferences, were measured with a graduated tape by trained personal. We have estimated the pattern of fat distribution by dividing waist circumference squared to weight (WSWT) which was adapted of Lutsey et al. [16].

### 2.2. Methods

Blood samples were collected from each participant, and genomic DNA was extracted using a commercial DNA extraction kit (QIAamp Blood Kit, Qiagen, Valencia, CA, USA). Genotypes for *ADIPOQ* (rs17366568 and rs182052), *RARRES2* (rs17173608 and rs4721), and *PPARGC1* (rs8192678 and rs3736265) variants were determined using real-time polymerase chain reaction TaqMan® assays (ThermoFisher, Carlsbad, CA, USA). Amplification was carried out in an ABI Prism 7500 Real-Time PCR System (ThermoFisher). All plates were included negative (all components excluding DNA) and positive internal controls for the genotyping quality conformation. There was 100% consistency in a 30% sample of duplicating test.

### 2.3. Statistical Analyses

Normality of continuous variables was tested by the Kolmogorov-Smirnov and Shapiro Wilk tests. Differences in continuous and categorical variables between two groups were calculated by the Mann–Whitney and *χ*^2^ tests, respectively.

Genotype and allele frequencies were estimated by gene counting. Genotype frequencies for each single nucleotide polymorphism (SNP) were tested for Hardy-Weinberg equilibrium using the *χ*^2^ test. Allele and genotype frequencies between control and case groups were compared by logistic regression, and the odds ratio (OR) was calculated. Differences on the genotype frequency between groups were carried out in dominant and recessive models. Log-transformation of anthropometric variables was performed before linear regression. The traits were chosen as the dependent variable, and the genotypes were tested as the independent variable (additive model). All regressions were adjusted for gender and age. The analyses were performed using the SPSS statistical package (IBM, Chicago, IL, USA). A *P* value less than 0.05 was considered statistically significant.

Sample size was calculated using an iterative process to compute the sample needed to test the difference between the two groups of a qualitative variable [17]. A conservative and convenience sample was chosen, since different polymorphisms were analyzed in this study (80% of statistical power).

## 3. Results

### 3.1. Basic Patient Characteristics

Anthropometric and biological variables of the 332 subjects, divided into cases and controls according to body mass index (BMI) status, are shown in Table 1. As expected, individuals with obesity had higher values of body measures than the control group. The exception was in height, which the control group had higher values.

### 3.2. Genotyping of Patient Samples Related to the Increased Risk for Obesity

In the present study, all six tested SNPs were polymorphic (minor allele frequency > 0.01). The sample was stratified by BMI, and details about the genotype and allele frequencies are presented in Table 2. Only SNP rs17366568 was not in Hardy-Weinberg equilibrium, both for cases (*P* < 0.001) and controls (*P* < 0.001). Therefore, rs17366568 was excluded in the following analyses.

Genotype frequencies of rs182052 in *ADIPOQ* were differed between the groups. Genotype AA was associated with obesity when compared to GG (*P* = 0.014), even after adjusted for gender and age (*P* = 0.023). In order to explore this association, we carried out the dominant and recessive models. The rs182052 was significantly associated with obesity in both models. However, after adjustment, this polymorphism was only related to obesity in a recessive model (*P* = 0.049). Furthermore, the allelic test showed that the minor allele frequency (A) was significantly higher in subjects with obesity than in controls (38.9 versus 29.4%). Therefore, individuals carrying the A allele had an increased risk for obesity (OR = 1.53 [1.11–2.11]). We did not find associations between SNPs in *RARRES2* and *PPARGC1* and obesity.

### 3.3. Associations between SNPs and Anthropometric Data

We investigated the impact of the SNPs on anthropometric variables. *ADIPOQ* rs182052 was associated with body weight (*P* = 0.009), BMI (*P* = 0.022), waist circumference (*P* = 0.005), and WSWT values (*P* = 0.034), after adjusting for gender and age (Table 3). Individuals carrying the A allele had higher body weight, BMI, waist circumference, and WSWT values than those homozygous for the G allele. None of the SNPs tested were associated with the waist-to-hip ratio (WHR).

Subsequently, the sample was stratified by BMI, and the analyses were made separately in the case and control groups. In the control subjects, the median body weight (*P* = 0.019) and waist circumference (*P* = 0.014) were different between the rs182052 genotype groups and the presence of the minor allele (A) was associated with higher progressive measurements (Table 4). In the case group, no association was found between any SNP and the anthropometric traits studied (data not shown).

Additionally, in individuals with morbid obesity (BMI ≥ 40.0), associations were found between *ADIPOQ* rs182052 and body weight (*P* = 0.006), BMI (*P* = 0.002), and waist circumference (*P* = 0.013) (Table 4).

We did not find associations between SNPs in *RARRES2* and *PPARGC1*, and anthropometric variables, either before or after stratification.

## 4. Discussion

In this study, *ADIPOQ* rs182052 was associated with obesity susceptibility. The AA genotype increased the risk for obesity when compared with genotype GG. Additional analysis showed that polymorphism is associated with obesity in recessive model. Moreover, our results showed that individuals carrying the A allele were more susceptible to obesity when compared to those with the wild-type allele. A previous study reported a similar result in Korean women, in which the A allele was associated with an increased risk for obesity [18].

There were some associations between SNPs and anthropometric characteristics. Our results showed a dose-effect relationship between *ADIPOQ* rs182052 and obesity measurements. We found that subjects with genotypes AA or AG had higher body weight, BMI, waist circumference, and WSWT values after adjustment for gender and age. Furthermore, when stratified by BMI, there were significantly higher values of body weight and waist circumference in control subjects carrying the A allele. In the entire group with obesity, we failed to detect any associations between the variables tested. However, increased body weight, BMI, and waist circumference were found in individuals with morbid obesity carrying the same risk allele. These results showed that the rs182052 polymorphism was associated with anthropometric traits in control and individuals with morbid obesity, but not the entire sample with obese people. There is no clear explanation for this result. However, it may be due to the limited sample size of obesity class I (30.0 ≤ BMI ≤ 34.9) and II (35.0 ≤ BMI ≤ 39.9) individuals.

Previous studies reported an association between rs182052 and obesity-related traits. Associations found in the current study are in agreement with Sutton et al. [19] who showed that the minor allele (A) was associated with higher BMI and waist circumference values in a Hispanic population. Similarly, Richardson et al. [20] found that the rs182052 A allele in Mexican individuals was significantly associated with an increased BMI. In the studies of Korean inhabitants, the same allele was related to increased body weight, WHR, and BMI in women [18]. In contrast, we did not find an association between this polymorphism and WHR. Interestingly, in a study on a cohort of Caucasian Americans, researchers found an interaction between rs182052, waist circumference, and levels of adiponectin. The Caucasian subjects with genotype AA had a reduction of 15.6% of serum adiponectin and an increased waist circumference when compared with those of genotype GG [21]. In another study, Hivert et al. [22] did not find an association between this polymorphism and BMI and waist circumference in a large cohort of European descent. In addition, a family-based study from a genetically isolated rural population of the Netherlands did not show a relationship between rs182052 and BMI or waist circumference [23]. These conflicting results may be attributable to differences in genetic background and different sample criteria, or other environmental factors [24].

The contribution of adiponectin to obesity is not well understood, but some studies have shown that individuals with obesity have lower plasma concentration of this protein than subjects without obesity [25, 26]. In addition, *ADIPOQ* is very polymorphic and several SNPs are associated with adiponectin levels [22, 27–29]. The rs182052 SNP is located in the first intron of *ADIPOQ*. However, the first intron of human *ADIPOQ* also contains a gene expression enhancer element, responsible for increasing the activity of its promoter [30]. Variants in this promoter region affect circulating adiponectin levels by modifying *ADIPOQ* expression. Thus, hypoadiponectinemia has been observed consistently with obesity [25, 26]. Based on these findings, we suggest that the *ADIPOQ* rs182052 polymorphism may be affecting adiponectin serum levels in our subjects and may disrupt energy balance by reducing energy expenditure [31]. This disruption, associated with an obesogenic environment, could result in increased body adiposity. However, replication and validation studies are necessary to confirm the role of *ADIPOQ* in the genetic susceptibility to obesity in different populations.

In this study, we have used a well-characterized cohort of participants, with or without obesity. Our sample has a significant portion of morbidly obese subjects, which are more difficult to recruit. However, one limitation of this study came from the cross-sectional study design, since we are not able to consider the fluctuation of body weight and other measurements over time. Furthermore, we are not able to fully isolate environmental factors, which could have an influence on the outcome.

In conclusion, our case-control study made in a southeastern Brazilian population suggests that the rs182052 polymorphism of *ADIPOQ*, but not the other SNPs tested, is associated with an increased risk for obesity and obesity-related parameters.

## Figures and Tables

**Table 1:** Subject characteristics.

	All	Control (18.5 ≤ BMI ≤ 24.9)	Case (BMI ≥ 30.0)	*P*
Age (years)	35 (27; 46)	30 (25; 38)	40 (31; 52)	<0.001
Gender (female/male)	231/101	98/67	133/34	<0.001
Weight (kg)	82.2 (63.0; 120.8)	63.0 (57.0; 70.3)	120.0 (102.4; 141.5)	<0.001
Height (m)	1.65 (1.60; 1.72)	1.69 (1.62; 1.74)	1.63 (1.58; 1.69)	<0.001
BMI (kg/m^2^)	30.1 (22.9; 45.3)	22.8 (21.0; 24.0)	45.3 (39.1; 51.6)	<0.001
Waist circumference (cm)	101.5 (84.9; 131.3)	85.0 (76.5; 93.5)	131.0 (118.4; 144.5)	<0.001
Hip circumference (cm)	107.8 (94.4; 140.0)	94.5 (84.0; 100.0)	140.0 (127.8; 151.0)	<0.001
WHR	0.93 (0.84; 1.02)	0.87 (0.80; 1.08)	0.95 (0.90; 1.01)	<0.001
WSWT	144.8 (130.5; 158.1)	97.9 (111.2; 129.0)	144.8 (130.5; 150.0)	<0.001

Data are presented as median values (interquartile range) for continuous traits and *n* (%) for categorical traits. BMI: body mass index; WHR: waist-hip ratio; WSWT: waist circumference squared divided by weight. *P* values for differences between case and control subjects.

**Table 2:** Genotype and allele frequencies of control and case individuals and association with the risk of obesity.

Genes	Polymorphism		Case	Control	OR (95% CI)	*P*	*P* ^¥^
			*n* = 167 (%)	*n* = 165 (%)			
*ADIPOQ*	rs182052						
	*Genotype*	GG	61 (36.5)	80 (48.5)	1.00 (Ref.)	—	—
		GA	82 (49.1)	73 (44.2)	1.47 (0.93–2.32)	0.098	0.179
		AA	24 (14.4)	12 (7.3)	2.57 (1.22–5.42)	**0.014**	**0.023**
		Dominant model					
		GG	61 (36.5)	80 (48.5)	1.00 (Ref.)	—	—
		GA + AA	106 (63.5)	85 (51.5)	1.63 (1.05–2.52)	**0.028**	0.061
		Recessive model					
		GG + GA	143 (85.6)	153 (92.7)	1.00 (Ref.)	—	—
		AA	24 (14.4)	12 (7.3)	2.14 (1.03-4.44)	**0.041**	**0.049**
	*Allele*	G	204 (61.1)	233 (70.6)	1.00 (Ref.)	—	—
		A	130 (38.9)	97 (29.4)	1.56 (1.12–2.19)	**0.009**	**0.020**

*RARRES2*	rs17173608						
	*Genotype*	TT	140 (83.8)	137 (83.0)	1.00 (Ref.)	—	—
		TG	24 (14.4)	27 (16.4)	0.87 (0.48–1.58)	0.648	0.191
		GG	3 (1.8)	1 (0.6)	2.94 (0.30–28.57)	0.354	0.483
		Dominant model					
		TT	140 (83.8)	137 (83.0)	1.00 (Ref.)	—	—
		TG + GG	27 (16.2)	28 (17.0)	0.94 (0.53–1.68)	0.844	0.272
		Recessive model					
		TT + TG	164 (98.2)	164 (99.4)	1.00 (Ref.)	—	—
		GG	3 (1.8)	1 (0.6)	3.00 (0.31–29.14)	0.344	0.364
	*Allele*	T	304 (91.0)	301 (91.2)	1.00 (Ref.)	—	—
		G	30 (9.0)	29 (8.8)	1.02 (0.61–1.72)	0.932	0.420
	rs4721						
	*Genotype*	TT	43 (25.7)	56 (33.9)	1.00 (Ref.)	—	—
		TG	91 (54.5)	81 (49.1)	1.46 (0.89–2.41)	0.134	0.405
		GG	33 (19.8)	28 (17.0)	1.54 (0.81–2.92)	0.190	0.873
		Dominant model					
		TT	43 (25.7)	56 (33.9)	1.00 (Ref.)	—	—
		TG + GG	124 (74.3)	109 (66.1)	1.48 (0.92–2.38)	0.104	0.481
		Recessive model					
		TT + TG	134 (80.2)	137 (83.0)	1.00 (Ref.)	—	—
		GG	33 (19.8)	28 (17.0)	1.21 (0.69–2.10)	0.512	0.774
	*Allele*	T	177 (53.0)	183 (57.2)	1.00 (Ref.)	—	—
		G	157 (47.0)	137 (42.8)	1.26 (0.92–1.74)	0.145	0.756

*PGC1a*	rs8192678						
	*Genotype*	GG	91 (54.5)	84 (50.9)	1.00 (Ref.)	—	—
		GA	62 (37.1)	71 (43.0)	0.81 (0.51–1.27)	0.350	0.519
		AA	14 (8.4)	10 (6.1)	1.29 (0.55–3.01)	0.561	0.681
		Dominant model					
		GG	91 (54.5)	84 (50.9)	1.00 (Ref.)	—	—
		GA + AA	76 (45.5)	81 (49.1)	0.87 (0.56–1.33)	0.513	0.657
		Recessive model					
		GG + GA	153 (91.6)	155 (93.9)	1.00 (Ref.)	—	—
		AA	14 (8.4)	10 (6.1)	1.41 (0.61–3.29)	0.416	0.566
	*Allele*	G	244 (73.1)	239 (72.4)	1.00 (Ref.)	—	—
		A	90 (26.9)	91 (27.6)	0.97 (0.69–1.37)	0.855	0.978
	rs3736265						
	*Genotype*	GG	151 (87.3)	144 (90.4)	1.00 (Ref.)	—	—
		GA	15 (11.5)	19 (9.0)	0.75 (0.37–1.54)	0.436	0.674
		AA	1 (1.2)	2 (0.6)	0.48 (0.04–5.32)	0.547	0.163
		Dominant model					
		GG	151 (90.4)	144 (87.3)	1.00 (Ref.)	—	—
		GA + AA	16 (9.6)	21 (12.7)	0.73 (0.37–1.45)	0.364	0.460
		Recessive model					
		GG + GA	166 (99.4)	163 (98.8)	1.00 (Ref.)	—	—
		AA	1 (0.6)	2 (1.2)	0.49 (0.44–5.47)	0.563	0.166
	*Allele*	G	317 (94.9)	307 (93.0)	1.00 (Ref.)	—	—
		A	17 (7.0)	23 (5.1)	0.74 (0.40–1.37)	0.333	0.312

*P* values for logistic regression. *P*^¥^ values adjusted for gender and age.

**Table 3:** Correlations of *ADIPOQ* rs182052 with anthropometric traits in the total patient sample.

Parameters	*ADIPOQ* rs182052			*P*
	GG	GA	AA	
	(*n* = 141)	(*n* = 155)	(*n* = 36)	
Age (years)	34.0 (27.0; 42.0)	36.0 (28.0; 47.0)	33.0 (28.0; 50.0)	0.323
Weight (kg)	72.0 (59.9; 116.4)	83.0 (65.2; 125.0)	92.0 (71.9; 127.2)	**0.009**
BMI (kg/m^2^)	24.7 (22.06; 44.20)	30.7 (22.92; 46.72)	34.9 (24.3; 49.1)	**0.022**
Waist circumference (cm)	98.0 (81.0; 130.00)	103.0 (85.8; 133.5)	111.8 (96.0; 139.5)	**0.005**
Hip circumference (cm)	102.0 (92.3; 134.0)	110.0 (95.8; 142.0)	124.0 (100.9; 142.5)	0.204
WHR	0.92 (0.82; 1.01)	0.93 (0.84; 1.02)	0.94 (0.89; 0.99)	0.349
WSWT	123.0 (130.1; 147.3)	133.0 (112.4; 153.3)	133.5 (117.4; 158.0)	**0.034**

Data are present as median values (interquartile range). BMI: body mass index; WHR: waist-hip ratio; WSWT: waist circumference squared divided by weight. *P* values for linear regression. Age was adjusted for gender. All obesity-related traits were adjusted for gender and age.

**Table 4:** Associations of *ADIPOQ* rs182052 with anthropometric variables in control individuals and subjects with morbid obesity.

Parameters	Control (18.5 ≤ BMI ≤ 24.9)			*P*	Case (BMI ≥ 40.0)			*P*
	GG	GA	AA		GG	GA	AA	
	(*n* = 80)	(*n* = 73)	(*n* = 12)		(*n* = 47)	(*n* = 61)	(*n* = 12)	
Age (years)	28.0 (24.0; 38.0)	31.0 (26.0; 39.0)	31.0 (21.0; 45.0)	0.619	39.0 (33.0; 44.0)	31.0 (42.0; 52.0)	30.0 (26.0; 33.0)	0.145
Weight (kg)	61.9 (56.0; 70.0)	63.7 (56.9; 70.5)	65.2 (61.2; 73.6)	**0.019**	131.5 (114.0; 138.0)	132.5 (117.7; 155.4)	144.8 (125.6; 183.5)	**0.006**
BMI (kg/m^2^)	22.6 (20.6; 24.1)	22.8 (21.2; 23.8)	23.3 (22.6; 24.5)	0.186	47.6 (44.1; 51.0)	48.5 (44.4; 57.4)	51.7 (47.7; 60.0)	**0.002**
Waist circumference (cm)	83.0 (74.3; 89.0)	85.5 (80.5; 94.0)	93.0 (78.1; 98.0)	**0.014**	138.0 (127.0; 145.5)	141.8 (128.0; 153.0)	142.5 (136.5; 155.5)	**0.013**
Hip circumference (cm)	94.0 (84.0; 99.0)	95.0 (83.0; 101.8)	97.5 (83.7; 101.6)	0.484	143.0 (134.0; 150.0)	147.0 (134.1; 158.0)	150.5 (141.5; 167.5)	0.998
WHR	0.85 (0.78; 1.04)	0.88 (0.82; 1.14)	0.94 (0.78; 1.19)	0.305	0.97 (0.91; 1.0)	0.97 (0.91; 1.01)	0.95 (0.91; 1.03)	0.539
WSWT	108.6 (96.0; 120.8)	113.4 (100.0; 134.0)	119.0 (93.9; 130.8)	0.082	145.7 (131.8; 161.0)	147.3 (133.0; 155.8)	146.4 (140.8; 164.0)	0.571

Data are present as median values (interquartile range). BMI: body mass index; WHR: waist-hip ratio; WSWT: waist circumference squared divided by weight. *P* values for linear regression. Age was adjusted for gender. All obesity-related traits were adjusted for gender and age.
